# Digital quantitative tissue image analysis of hypoxia in resected pancreatic ductal adenocarcinomas

**DOI:** 10.3389/fonc.2022.926497

**Published:** 2022-08-01

**Authors:** Iram Siddiqui, Jade Bilkey, Trevor D. McKee, Stefano Serra, Melania Pintilie, Trevor Do, Jing Xu, Ming-Sound Tsao, Steve Gallinger, Richard P. Hill, David W. Hedley, Neesha C. Dhani

**Affiliations:** ^1^ Department of Pediatric Laboratory Medicine, The Hospital for Sick Children, Toronto, ON, Canada; ^2^ Spatio-temporal Targeting and Amplification of Radiation Response (STTARR), University Health Network, Toronto, ON, Canada; ^3^ Department of Pathology, Toronto General Hospital, Toronto, ON, Canada; ^4^ Department of Biostatistics, The Princess Margaret Cancer Centre, Toronto, ON, Canada; ^5^ Department of Medical Oncology, The Princess Margaret Cancer Centre, Toronto, ON, Canada; ^6^ PanCuRx Translational Research Initiative, Ontario Institute for Cancer Research, Toronto, ON, Canada; ^7^ Hepato-Pancreatico-Biliary Surgical Oncology Program, University Health Network, Toronto, ON, Canada; ^8^ Medicine Program, The Princess Margaret Cancer Centre/Ontario Cancer Institute, Radiation Toronto, ON, Canada

**Keywords:** hypoxia, tumor microenvironment, ductal adenocarcinoma (PDAC), tumor heterogeneity, image analysis

## Abstract

**Background:**

Tumor hypoxia is theorized to contribute to the aggressive biology of pancreatic ductal adenocarcinoma (PDAC). We previously reported that hypoxia correlated with rapid tumor growth and metastasis in patient-derived xenografts. Anticipating a prognostic relevance of hypoxia in patient tumors, we developed protocols for automated semi-quantitative image analysis to provide an objective, observer-independent measure of hypoxia. We further validated this method which can reproducibly estimate pimonidazole-detectable hypoxia in a high-through put manner.

**Methods:**

We studied the performance of three automated image analysis platforms in scoring pimonidazole-detectable hypoxia in resected PDAC (n = 10) in a cohort of patients enrolled in PIMO-PANC. Multiple stained tumor sections were analyzed on three independent image-analysis platforms, Aperio Genie (AG), Definiens Tissue Studio (TS), and Definiens Developer (DD), which comprised of a customized rule set.

**Results:**

The output from Aperio Genie (AG) had good concordance with manual scoring, but the workflow was resource-intensive and not suited for high-throughput analysis. TS analysis had high levels of variability related to misclassification of cells class, while the customized rule set of DD had a high level of reliability with an intraclass coefficient of more than 85%.

**Discussion:**

This work demonstrates the feasibility of developing a robust, high-performance pipeline for an automated, quantitative scoring of pimonidazole-detectable hypoxia in patient tumors.

## Background

Histopathological tumor analysis has historically been the foundation of cancer diagnosis and prognostication. In the pursuit of targeted treatment approaches, a number of molecular analyses have now become standard pathological assays, with the most extensively used being the immunohistochemical (IHC) detection of proteins. In spite of its widespread use, however, IHC can be confounded by resource-intensive analysis (1), and poor inter-laboratory, inter-observer, and intra-observer reproducibility (2, 3). Several factors contribute to the variability of the output of IHC analyses, including the selection of appropriate tumor regions, heterogeneity in marker expression, variations in antibody performance and staining techniques, and the subjectivity and qualitative nature of traditional manual scoring (4).

Several complementary strategies have been recommended to improve on the stringency of IHC tumor analysis. These include robust guidelines around optimization of IHC staining methods, including the use of automation (5) and considerations on the issue of marker heterogeneity, as has been studied by ourselves and others (6). Finally, the validation and adoption of automated digital image analysis have the potential to provide IHC tumor analysis with the objectivity, reliability, and speed required for effective biomarker research with translation to the clinic (4). Several independent groups have already demonstrated at least equal, if not superior, performance of automated digital image analysis (DIA) versus traditional manual scoring (7, 8).

In the context of tumor hypoxia, we recently completed the quantitative scoring of pimonidazole IHC in a cohort of resected pancreatic ductal adenocarcinomas (PDAC) in patients accrued to the PIMO-PANC trial, using the adaptive, pattern-recognition, image analysis platform, Aperio Genie (1). Pimonidazole (1-[(2-hydroxy-3-piperidinyl) propyl]-2-nitromidazole hydrochloride) is an exogenous hypoxia tracer with an extensive prior use in preclinical and clinical hypoxia studies and is a well-established technique for assessing tissue hypoxia (9–11). This 2-nitroimidazole undergoes bioactive reduction to form covalent adducts with thiol-containing macromolecules in hypoxic (pO_2_ < 10 mmHg) but metabolically viable cells (12, 13). Adducts are then identified using different immune-detection methods including IHC. Pimonidazole studies have historically utilized a semiquantitative, ordinal scoring system (14), which remains susceptible to bias and variability, given its basis in manual visual scoring. Another relevant limitation of ordinal scoring systems is the potential for non-linear relationships across categories, to confound correlation with biological data. The work we describe here was initiated with the primary objective of developing and validating a pipeline for image analysis that was (1) reproducible, (2) relatively user-independent, and (3) could be applied in a high-throughput manner. Further, given the emerging contributions of cancer-associated stromal cells to tumor biology and clinical behavior, we wanted a method that would be able to confidently differentiate between tumor epithelial and stromal cellular compartments (15, 16).

In our initial analysis, Aperio Genie provided a quantitative, and continuous, estimate of tumor hypoxia that had good concordance with manual scoring; analysis of five full tumor sections per each patient tumor was able to appropriately account for tumoral heterogeneity (1). However, the workflow was quite resource-intensive, with each tumor requiring its own customized analysis algorithm and settings. Further, distinguishing epithelial from stromal cells was challenging on the pixel-based Genie platform. We therefore proceeded to evaluate two other image analysis platforms that were in common use at our institution, both of which better resolve distinct cell types through improved cell segmentation algorithms. Definiens Tissue Studio utilizes a prepackaged, generic cellular segmentation methodology, with limited adaptability, while Definiens Developer allows customized modifications of cellular segmentation. We describe here the results of our comparison of quantitative tumor image analysis of pimonidazole IHC on these three platforms.

## Materials and methods

### Study details

PIMO-PANC (NCT01248637) is a prospective, REB-approved, single-institution trial conducted at the Princess Margaret Cancer Centre/University Health Network. Eligible patients were 18 years or older, being considered for surgery with a presumed diagnosis of localized pancreatic ductal adenocarcinoma (PDAC). The primary objective is to evaluate the effect of hypoxia on survival of early-staged (resectable) PDAC.

Registered patients received a single dose of the hypoxia marker pimonidazole, on the day prior to surgery. Resected tumors were evaluated and processed as per institutional standard practice for a clinical diagnosis. All archived hematoxylin and eosin (H&E)-stained slides were retrospectively reviewed by an expert GI pathologist, and at least five representative tumor sections were identified. Tumor tissue selection criteria included sections containing viable tumor occupying the most surface area with minimal artifacts (such as necrosis and variations in tissue processing). Sections were cut (4 µm) from the five selected tumor blocks for pimonidazole immunohistochemical (IHC) staining and analysis with Aperio Genie (Ref 1) and Tissue Studio. Subsequently, new sections were cut and stained for PIMO immunohistochemistry from the same tumor blocks for Definiens Developer analysis. Data from Aperio Genie were used from the previous study (1), for comparison.

### Pimonidazole immunohistochemistry protocol

FFPE tumor sections were dried at 60°C for 1–2 h and IHC staining completed as per the manufacturer’s guidelines, using an automated slide stainer (BenchMark XT, Ventana Medical Systems) with medium antigen retrieval (CC1, Tris/borate/EDTA pH 8.0, #950-124). The dilution for pimonidazole antibody (Hypoxyprobe, Inc.) was 1:400, with incubation time of 60 min. Secondary detection was completed using Ventana ultraview Universal DAB Detection Kit (#760-500) and visualization by hydrogen peroxide substrate and 3,3′-diaminobenzidine tetrahydrochloride (DAB) chromogen. Slides were counterstained with Harris hematoxylin and Bluing in PBS, dehydrated in graded alcohol, cleared in xylene, and coverslipped in Permount. Stained sections were digitized for analysis (Aperio ScanScope, Leica Biosystems Inc., Carlsbad CA).

### Quantitative image analysis

Pimonidazole IHC-stained tumor slides from 10 patients were scanned and analyzed on three independent image-analysis platforms as outlined below.

### Aperio genie

Analysis was completed as described previously (1). Briefly, regions of interest (ROIs) were manually annotated on scanned images of the IHC-stained tumor slides for analysis, excluding areas of necrosis and non-neoplastic normal tissue adjacent to tumor. Classes “epithelium,” “stroma,” and “other” were defined and used to develop unique classifiers for each patient tumor to differentiate epithelial from stromal tumor compartments. The “other” class was used to define regions to exclude from analysis (e.g., necrosis, non-pancreatic tissue). Aperio’s Positive Pixel v9 algorithm was applied to quantify hypoxic percentages (HPs) in epithelial and stromal tumor compartments (with HP-whole tumor = HP-epithelial + HP-stromal) within annotated ROIs.

### Definiens tissue studio

All scanned slide images of the of the IHC-stained tumor slides were loaded into Tissue Studio (TS) 4.0 (Definiens Inc., Munich, Germany). A machine learning classifier differentiating “stroma” from “epithelium” was developed by providing examples of images of both tissue classes, as well as tissue artifact to be excluded from analysis using the decision tree algorithm. This classifier was then applied to refine regions of interest, followed by a pathologist review and manual correction of any regions incorrectly labeled by the automated classifier including manual extraction of background normal tissue. It should be noted that the manual correction was performed individually on every image from each tumor.

A stain separation algorithm was used to separate hematoxylin from the DAB signal, with nuclear segmentation being performed based on the hematoxylin signal. Cell size was estimated and simulated by growing an area of cytoplasm 2 microns from every nucleus. A threshold applied to the intensity of the DAB signal was used to differentiate between pimonidazole-positive and -negative cellular regions.

### Definiens developer XD

Scanned images were manually annotated by the study pathologist to select tumor regions only. At this time, any large areas of necrosis within the tumor region were also excluded. This initial step of manual annotation of the tumor region approximately took an average of 5 min per image. A custom set of algorithms for cellular segmentation and classification was developed with direct input from a platform programmer and a study pathologist as outlined in detail below.

#### Development of custom classification algorithms

The white balance of respective slides was computed to correct for uneven lighting in slide scanning, and the DD stain separation algorithm was used to separate DAB and hematoxylin stains into unique image channels. Information regarding white balance and stain color coefficients was used to improve stain channel accuracy. Preliminary ROIs were then re-annotated to exclude whitespace and other obvious artifacts.

Fifty ROIs (512 × 384 microns) were randomly selected across slides from 68 patient tumors and divided into two groups. One group of 25 ROIs was designated as the *training set*, with the remaining 25 assigned as the *validation set*. Two expert GI pathologists used an open-source image editing program (GIMP, The GIMP Development Team, Retrieved from https://www.gimp.org) to independently manually annotate cells within all 50 fields as “epithelial”, “stromal”, or “inflammatory cell/other”, further differentiating pimonidazole-positive cells as “stained” and pimonidazole-negative cells as “unstained”. Cells annotated as inflammatory cell/other were excluded from hypoxia analysis. A “consensus annotation” methodology was used to resolve discrepancies between pathologists’ annotations. If both pathologists’ annotations agreed, or if one pathologist annotated a cell which the other did not, then the agreed or positive identification was assigned. In cases of epithelial/stromal mismatch, the cell was identified as stroma to reduce misclassification of non-epithelial cells as epithelial. Likewise, for inflammatory/other cell mismatch, any cell identified as such by one of the two pathologists was classified as inflammatory, to ensure stringency of epithelial cell discrimination.

A training set of 25 grids was used to iteratively develop a custom cell classifier algorithm with joint input from a study pathologist and a platform programmer. The hematoxylin channel was used to segment ROIs into nuclear (high hematoxylin signal) or cytoplasmic (low hematoxylin signal) segments. Nuclear segments were then classified as either “epithelial,” “stromal,” or “other” (which included primarily inflammatory cells) using a custom-trained, pixel-wise Random Forest classifier trained on the “consensus annotation” applied to each detected cell segment within the segmented 25 field training sets. Nuclear segments were expanded into cytoplasmic tissue segments to simulate epithelial cells, fibroblasts, and inflammatory cells/other cell bodies using cell-type-specific, sizing heuristics. Cellular segments were individually assessed using the information present in the DAB stain channel. Segments were designated pimonidazole positive if more than 50% of their mean optical density (commonly: opacity/translucency) was derived from the DAB channel (and the optical density was above a minimum threshold of 0.1). This approach was selected as it agreed with pathologist assessment of stain intensity and performed well independent of cellular density and stain concentration. Simulated cellular segments were then used to designate larger regions of tumor tissue as predominantly containing epithelial, stromal, or inflammatory cells/other. Stromal tissue regions were then further classified as “cellular” or “acellular” by subtracting the stromal cell segments based on the average size of a fibroblast and classifying the remaining stromal tissue area as “acellular.”

#### Cell classifier validation

**Figure 1:**
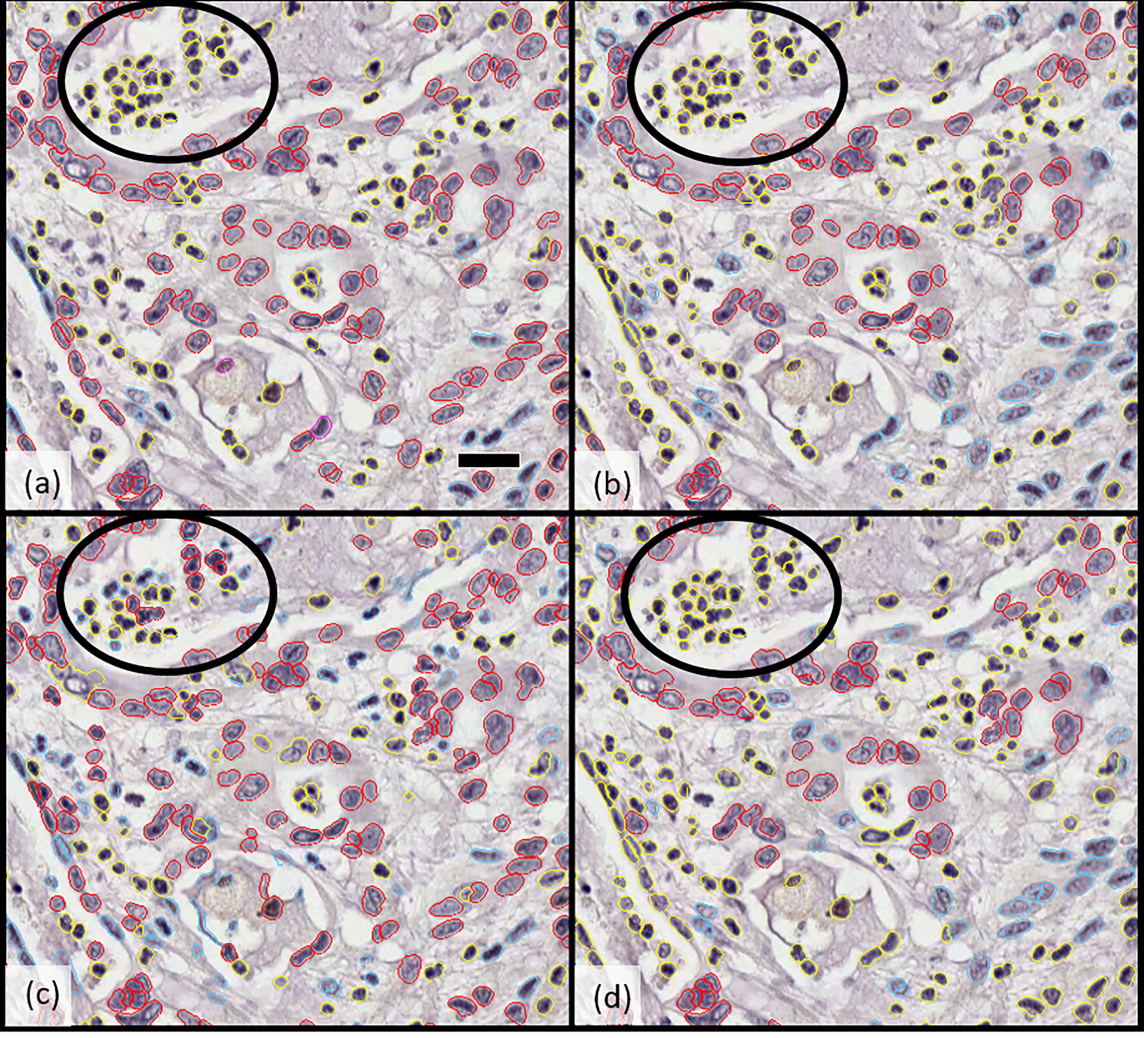
Comparison of cellular classification (epithelial vs. stromal) by **(A)** pathologist 1, **(B)** pathologist 2, **(C)** Developer algorithm v1, and **(D)** Developer algorithm v2 with epithelial cells highlighted in red and stromal cells in blue; cells excluded from analysis (including inflammatory cells, necrotic cells, and others) are indicated in yellow. Circled regions highlight the example of a region of cellular misclassification with cells identified as necrotic and coded yellow by both pathologists (to be excluded from analysis), which were classified as epithelial (red) or stromal (blue) by Developer algorithm v1. After further optimization and derivation of Developer algorithm v2, these cells were now excluded (highlight yellow). Scale bar in a) 25 microns.

The derived cell segmentation and classification algorithm was then applied to the 25 fields of the validation set to calculate concordance of classification between individual pathologists (IS and SS), combined-pathologist scoring (“joint”), and DD. There was greater reliability across the two pathologists’ scoring of epithelial cells (>72%) than stromal cells (>66%). When pathologists’ scoring was combined to define a “consensus annotation” or “joint classification,” the machine-based algorithm had an 86% alignment with manual scoring of epithelial tumor cells (**Figure 1**).

### Cross-platform comparison and statistical analysis

A cross-platform agreement of quantitative estimates of hypoxia was analyzed in a 10-patient tumor cohort. Spearman correlation coefficients were calculated to assess the concordance between the hypoxia level for the different techniques and between epithelial hypoxia and stromal hypoxia. Mixed-effect modeling was employed to obtain the variances between patients (inter-tumor heterogeneity) and within a patient (intra-tumor heterogeneity). Based on these variances, the intraclass correlation coefficients (ICC) were calculated. The ICC is a measure of reliability ranging from 0 to 1.0; values equal to or greater than 0.85 indicate a high level of reliability across measurements. These calculations were performed using all sections and all patients available (10 patient tumors for Aperio Genie and Definiens Tissue Studio analysis; 92 patient tumors for Definiens Developer). We have calculated the ICC corresponding to analysis on one section per patient tumor, as well as with two to five sections per tumor (**Table 1**). All analyses were performed utilizing R 3.4 software (https://cran.r-project.org/).

**Table 1:** Reliability of estimation of hypoxia using different platforms based on number of slides evaluated.

No. of tumour sections evaluated	Intra-class co-efficient (ICC)								
	Developer			Genie			Tissue Studio		
	HPwt	HPepi	HPstr	HPwt	HPepi	HPstr	HPwt	HPepi	HPstr
1	0.728	0.736	0.713	0.678	0.702	0.567	0.325	0.308	0.059
2	0.842	0.848	0.832	0.808	0.825	0.724	0.490	0.471	0.112
3	0.889	0.893	0.882	0.863	0.876	0.797	0.591	0.572	0.159
4	0.914	0.918	0.909	0.894	0.904	0.840	0.658	0.640	0.201
5	0.930	0.933	0.925	0.913	0.922	0.868	0.706	0.690	0.240

## Results

**Figure 2:**
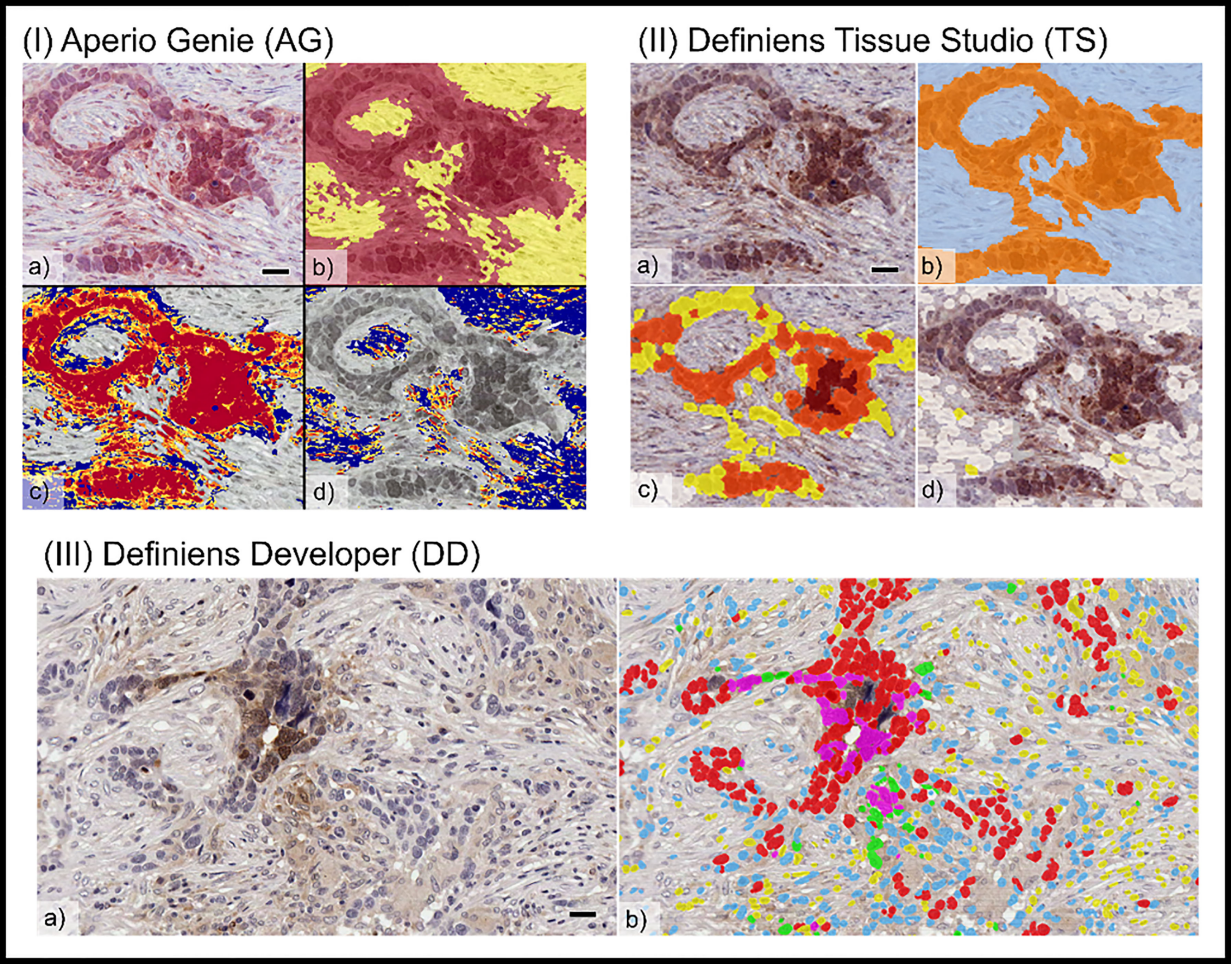
Comparison of tumor cellular classification with image overlay as processed on different image analysis platforms: (I) Aperio Genie: **(A)** pimonidazole (pimo), IHC **(B)** epithelial (red), from stroma (yellow) classification; **(C)** pimo +ve (red/orange) vs. pimo -ve (blue) in epithelial tumor; **(D)** pimo+ (red/orange) vs. pimo -ve (blue) in stromal tumor. (II) Definiens Tissue Studio: **(A)** pimonidazole IHC; **(B)** epithelial (orange) from stroma (light blue) classification; **(C)** pimo +ve (brown/orange/yellow) from pimo -ve (white) in epithelial compartment; **(D)** pimo+ (brown/orange/yellow) from pimo -ve (white) in stromal compartment; and (III) Definiens Developer: **(A)** pimonidazole IHC; **(B)** segmented cell overlay with pimo +ve epithelial cells (pink), pimo -ve epithelial cells (red), pimo +ve stromal cells (green), pimo -ve stromal cells (blue), inflammatory/other (yellow).

There were visible differences in the resolution of cellular classification across the three platforms related to the pixel-based segmentation algorithms utilized by Aperio Genie compared with the cell-based segmentation of Definiens Tissue Studio and Definiens Developer (**Figure 2**).

### Hypoxia is variable across patient tumors

Consistent with our previous reports, pimonidazole-detectable hypoxia is variable across patient tumors and appears to exist along a continuous spectrum (1). Hypoxia levels in epithelial tumor regions are concordant with levels in stroma as measured by all three image analysis platforms (Spearman’s coefficient 0.69 (Genie), 0.79 (DD), 0.88 (TS)).

**Figure 3:**
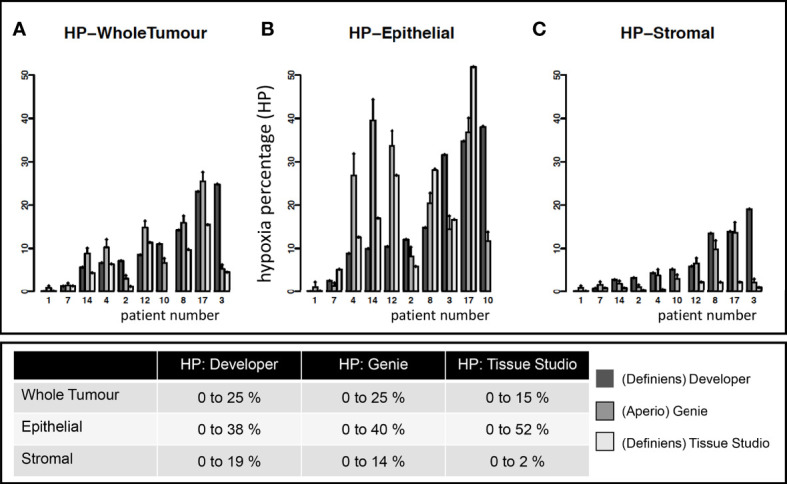
Estimates of tumoral hypoxia by different image analysis platforms. Each point on the x-axis represents a unique study patient. Each patient (except 10) has estimates of hypoxia on Definiens Developer, Genie, and Tissue Studio as indicated by different colored bars. Y-axis shows the hypoxia percentage (HP) (i.e., pimonidazole-detected hypoxia) in specific tumor compartments: **(A)** whole tumor, **(B)** epithelial, and **(C)** stromal tumor compartments.

Variability in quantitative estimates of hypoxia in 10 patient tumors using three different platforms is summarized in **Figure 3**. The range of whole-tumor HP was 0% to 26% as measured by both (Aperio) Genie and (Definiens) Developer and 0% to 15% as measured by (Definiens) Tissue Studio. Higher levels of hypoxia were observed in the epithelial tumor compartments, with estimates of HP-epithelial ranging from 0% to 38% by Developer, 0% to 40% by Genie, and 0% to 52% by Tissue Studio. Estimates of HP-stroma were unexpectedly low using Tissue Studio (0 to 2%) in comparison with the other two platforms (0% to 14% by Genie and 0% to 19% by Developer).

### Factors contributing to heterogeneity of quantitative hypoxia measurements

**Figure 4:**
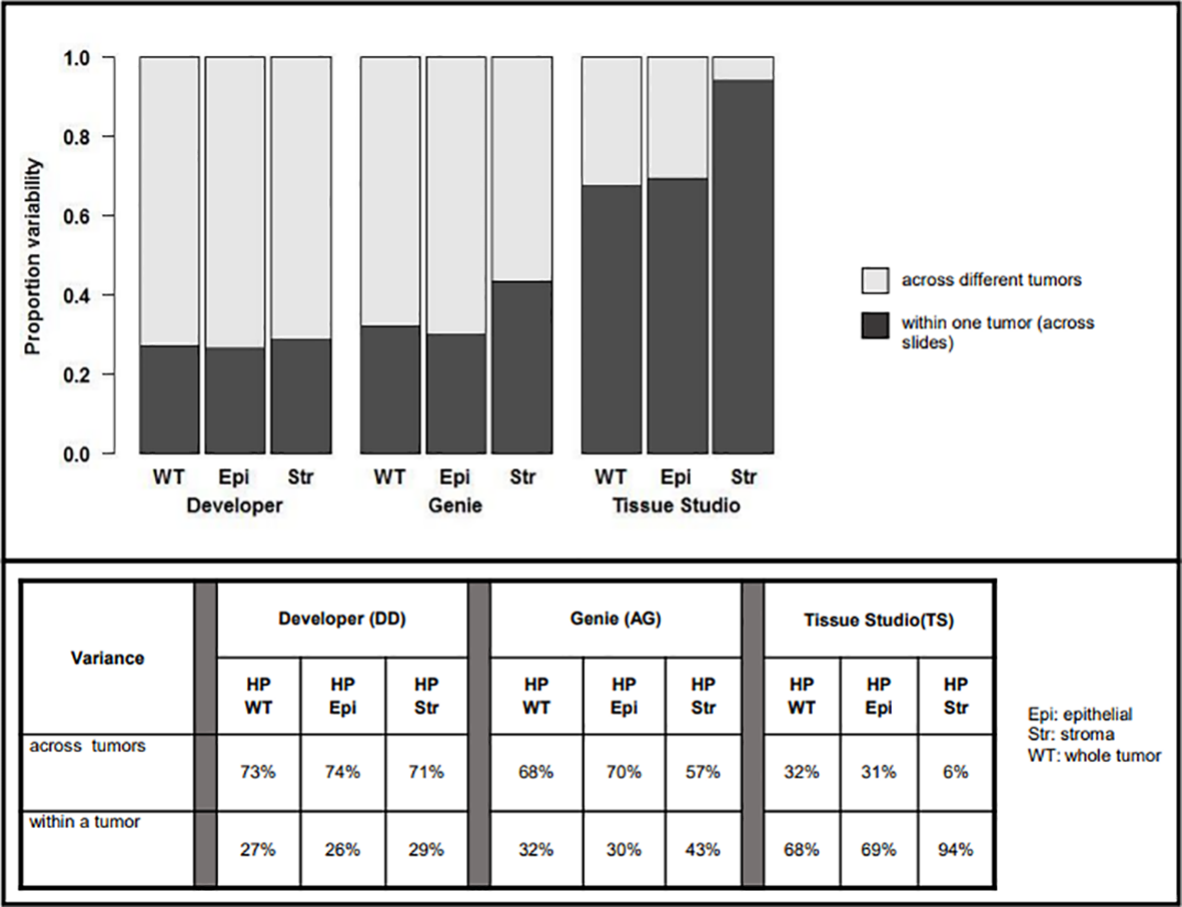
Variability in assessment of hypoxic percentage (in different tumor compartments) across platforms related to inter-patient (light gray) vs. intra-patient (dark grey) variability.

The respective contributions of intra- and interpatient heterogeneity to the variability of the estimates of HP were evaluated by mixed-effect modeling; these results are summarized in **Figure 4**.

For Developer and Genie analyses, most of the variability in measurement was related to heterogeneity across different tumors, with a lower proportion of the variability being related to the heterogeneity within a particular tumor. For example, with Developer analysis, 73% of the variability inherent in measures of HP-whole tumor was interpatient variance while 27% was related to heterogeneity within a tumor; for analysis on Genie, the numbers were 68% (inter-) vs. 32% (intra), respectively. The comparatively higher inter- vs. intra-patient heterogeneity on Genie and Developer suggests that automated image analysis (AIA) estimates of tumoral hypoxia using these platforms will identify real differences in HP across patients. In contrast, most of the variability inherent in the Tissue Studio analysis was related to heterogeneity within a tumor (for HP-whole tumor, 68% intra-patient vs. 32% interpatient heterogeneity). The high level of intra-patient heterogeneity reduces the confidence with which estimates of HP by Tissue Studio approximate “true” tumoral hypoxia, and the degree to which this analysis is likely to differentiate biologically real and relevant differences in hypoxia levels across patients is low.

Hypoxia was more variable in the stromal vs. epithelial tumor compartment on all three platforms—29% vs. 26% on Developer, 43% vs. 30% on Genie, and 94% vs. 69% on Tissue Studio. This suggests that HP-epithelial, with its relatively high inter-patient and low intra-patient heterogeneity, would be the best measure to use to differentiate among tumors based on levels of pimonidazole-detectable hypoxia.

**Figure 5:**
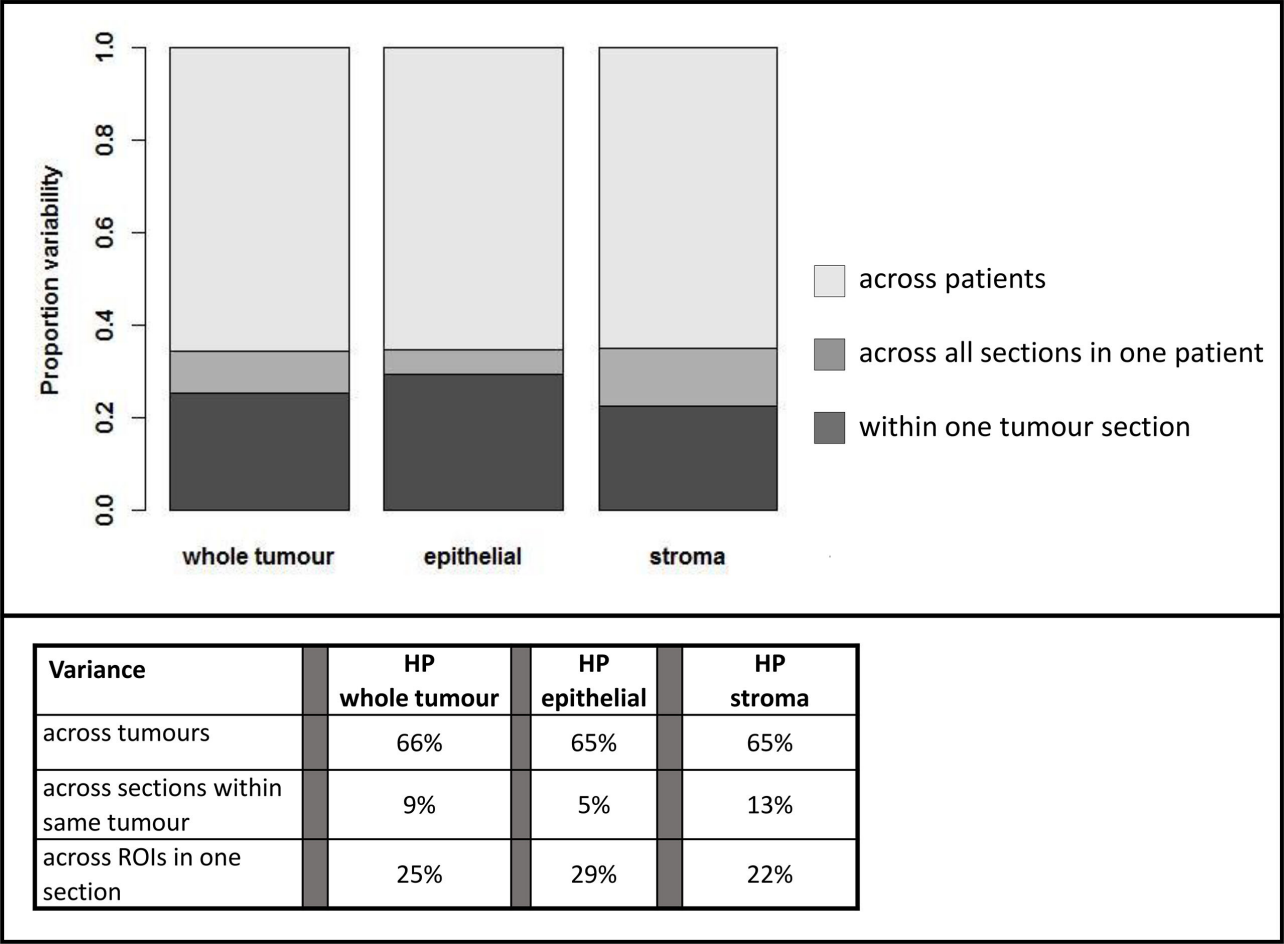
Estimation of variability of hypoxia estimated by Definiens Developer analysis within given patients and across patients. HP; hypoxic percentage.

An additional contributor to the heterogeneity of hypoxia measurements within a given patient’s tumor is the variance within and across slides. In the analysis completed on Developer, there was less variability across different sections of the same tumor compared with the variability within one section. For example, in estimating HP tumor, 25% of the variance inherent in the measure of hypoxia was related to variability across different ROIs in a given tumor section while 9% of the variance was related to variance across different sections and 66% of the variance in the measure was related to inter-patient variability (**Figure 5**).

### Reliability of estimates of HP based on number of sections evaluated

We calculated the intraclass correlation coefficient (ICC) using the mean of two or more values for each tumor, to understand the reliability of estimates of HP related to the number of tumor sections evaluated per patient tumor, with values equal to or greater than 0.85 indicating a high level of reliability across measurements. The results of this analysis are summarized in **Table 1**. As expected, ICC values increased with greater number of sections evaluated. Across the different platforms, calculated ICC was highest with Developer (0.88 for HP-stroma and 0.89 for both HP-epithelial and HP-whole tumor if three tumor sections were analyzed per patient tumor) and lowest with Tissue Studio (0.24 for HP-stroma, 0.69 for HP-epithelial, and 0.71 for HP-whole tumor if five sections were analyzed). These data suggest that analysis of three (representative) tumor sections would be sufficient to reliably estimate HP in resected pancreatic cancers using Developer but by contrast, analysis on Tissue Studio had high intra-patient variability, such that even evaluation of five tumor sections had poor reliability.

## Discussion

We present here our results studying three unique image-analysis platforms with computer-based learning capabilities, for their ability to provide quantitative estimates of pimonidazole-detectable hypoxia in surgically resected pancreatic cancers. These studies add to our prior work where an extensive, iterative training process was used to develop tumor-individualized scoring algorithms for the pixel-based platform Genie. This semiquantitative strategy provided estimates of pimonidazole tumor staining that were highly concordant with manual scoring (1). Its primary limitation, however, was the need to develop a customized algorithm for each tumor analyzed, resulting in a strategy that was cumbersome and impractical for high-throughput analysis. We have now compared these results with analyses conducted on two other image analysis platforms with different cellular segmentation capabilities—Developer and Tissue Studio. Both platforms were selected for study based on their contemporary use at our institution at the time and to test the hypothesis that automated analysis platforms performing tumor/stroma differentiation at a cellular level would yield more reproducible and accurate estimates of pimonidazole staining, which could be completed in a high-throughput manner.

We observed significant variability in pimonidazole staining both within and across patient tumors, using all three platforms. Quantitation by Developer and Genie were closely aligned, but estimates of stromal hypoxia by Tissue Studio were much lower than those made on the other two platforms. Pimonidazole scoring on both Developer and Genie had greater inter-patient than intra-patient heterogeneity, suggesting that either of these techniques should be able to confidently discern differences in levels of pimonidazole-detectable hypoxia across patients. In contrast, the high intra-patient variability of Tissue Studio hypoxia estimates compromises the utility of this platform to discriminate biologically relevant differences in hypoxia levels across patients.

The calculated intraclass coefficients (ICCs) provided insights into the impact of increasing the number of estimates made per patient tumor on measurement reproducibility, with an ICC of 0.85 (or 85%) considered good reliability. Analysis of three sections per patient tumor on Developer was sufficient for reliable estimates of both epithelial and stromal hypoxia, in comparison with the five required for analysis on Genie. This finding likely reflects the improved accuracy of cellular classification and differentiation of epithelial from stromal cells, using Developer with its cell-based segmentation, compared with the pixel-based platform Aperio. In contrast, the low ICC estimates of the Tissue Studio analysis underscore its low reliability in architecturally complex tissue like PDAC, likely due to the inferior epithelial/stromal discrimination by tissue-level classifiers. In Developer, we were able to develop customized, pathologist-guided, and cell-based segmentation algorithms that could use random-forest-based machine learning classifiers to identify unique cell phenotypes. In contrast, Tissue Studio analyses apply a generic, tissue-level classifier to differentiate epithelial from stromal regions, and subsequently, standard, computer-vision based, nuclear segmentation algorithms are used for cellular discrimination. Although this strategy does allow for a higher degree of cellular discrimination than pixel-based platforms like Genie, the complex architecture of PDAC tumor tissue meant that several rounds of refinement and manual correction were required, limiting both the consistency and throughput of this analysis.

Although the development of the Developer rule set was time/resource intensive, once optimized, the trained classifier could be applied across hundreds of slides, with the only manual intervention being a pre-analysis annotation of tumor regions of interest, a process that took few minutes per slide. This provided an efficient and reliable workflow with significant reduction in time spent for post-segmentation ROI correction. Following annotation, whole-slide processing utilizing a tiled approach enabled the analysis of 0.5–1 slides per hour on a desktop server running two Developer CPU engines simultaneously. The time requirement for these same tasks on platforms using tissue-level classifiers with manual correction was on average 20+ hours per image, highlighting additional advantages to the cellular segmentation-based analysis methods.

We recognize that since the completion of this work, several other digital image analysis platforms have emerged with comparable capabilities and more modern interfaces than those discussed here. A similar workflow in which cellular- or tissue-level features are used to build a segmentation strategy guided by input from expert disease-site pathologists should provide similar results. In future directions from this work, the cellular segmentation map output could also be leveraged as training data for more contemporary machine learning or AI-based image analysis approaches.

Although manual scoring by expert pathologists remains the standard method of immunohistochemical analysis, its robustness and broader applicability can be deeply affected by subjectivity and interobserver variability (17). Attempts to improve on between-pathologist reproducibility and within-pathologist repeatability has led to the exploration of field-of-view analysis (18). Further semiquantitative scoring systems have been derived to convert subjective descriptions of IHC-marker expression into quantitative data. One such tiered system was historically used in prior pimonidazole-based hypoxia scoring studies and performed well in comparison with manual scoring. However, the categorization of data results in loss of information that could be inferred from continuous variables, and unless category borders are well defined *a priori*, border misclassification introduces ambiguity in analysis (4). In the specific context of hypoxia scoring, a lack of clarity with respect to biologically relevant thresholds of pimonidazole-detectable hypoxia results in the use of arbitrary cut-points to define categories. All of these issues have the potential to obscure biologically relevant differences across tumors, limiting the utility of an analysis method. It is worth mentioning that, in spite of the clearly recognized prognostic significance of tumoral hypoxia, therapeutic targeting of this microenvironmental feature has been challenging, perhaps in part due the lack of robust tools for defining patient subgroups based on tumor hypoxia levels.

Contemporary platforms of image analysis with cellular segmentation capability, and utilizing computer-based learning algorithms for rule-set development, combines the discrimination power of manual scoring by expert pathologists, with the consistency and high throughput of automated digital pathology (19). Furthermore, the whole-section analysis that is possible with automated digital pathology appears to have greater reproducibility than field-of-view, manual scoring (20). There is the further advantage that computational analyses may have greater discriminatory power than human visual perception; however, whether there is biological relevance to these differences remains to be determined.

In conclusion, we have presented in this report our workflow and preliminary results from a quantitative, automated digital image analysis that can be applied to formalin-fixed, clinical PDAC tumors in a high-throughput manner. This method has been applied to the full dataset of PIMO-PANC patient tumors to explore relationships between hypoxia and prognosis in patients with early-stage, pancreatic ductal adenocarcinoma. In future work, we will be exploring the potential to modify the current algorithms, with the input of expert pathologists, for application to other tumor types.

## Data availability statement

The raw data supporting the conclusions of this article will be made available by the authors, without undue reservation.

## Ethics statement

The studies involving human participants were reviewed and approved by University Health Network. The patients/participants provided their written informed consent to participate in this study.

## Author contributions

IS, JB, TDM, TD carried out the research under the supervision of NCD and DWH. IS, ND and DWH planned all analysis. JB, IS, TD and TDM carried out the machine learning development and analysis. JX carried out histopathology technical support. IS, JB, TDM and NS wrote the paper with contributions from all authors. All authors approved the final manuscript.

## Conflict of interest

The authors declare that the research was conducted in the absence of any commercial or financial relationships that could be construed as a potential conflict of interest.

## Publisher’s note

All claims expressed in this article are solely those of the authors and do not necessarily represent those of their affiliated organizations, or those of the publisher, the editors and the reviewers. Any product that may be evaluated in this article, or claim that may be made by its manufacturer, is not guaranteed or endorsed by the publisher.
