# Supplementary material for: Digital quantitative tissue image analysis of hypoxia in resected pancreatic ductal adenocarcinomas
Source: Front Oncol. 2022 Aug 1;12:926497. doi: 10.3389/fonc.2022.926497 (PMC9376475; doi:10.3389/fonc.2022.926497)
Supplement: Supplementary file 1 [file Image_1.pdf]

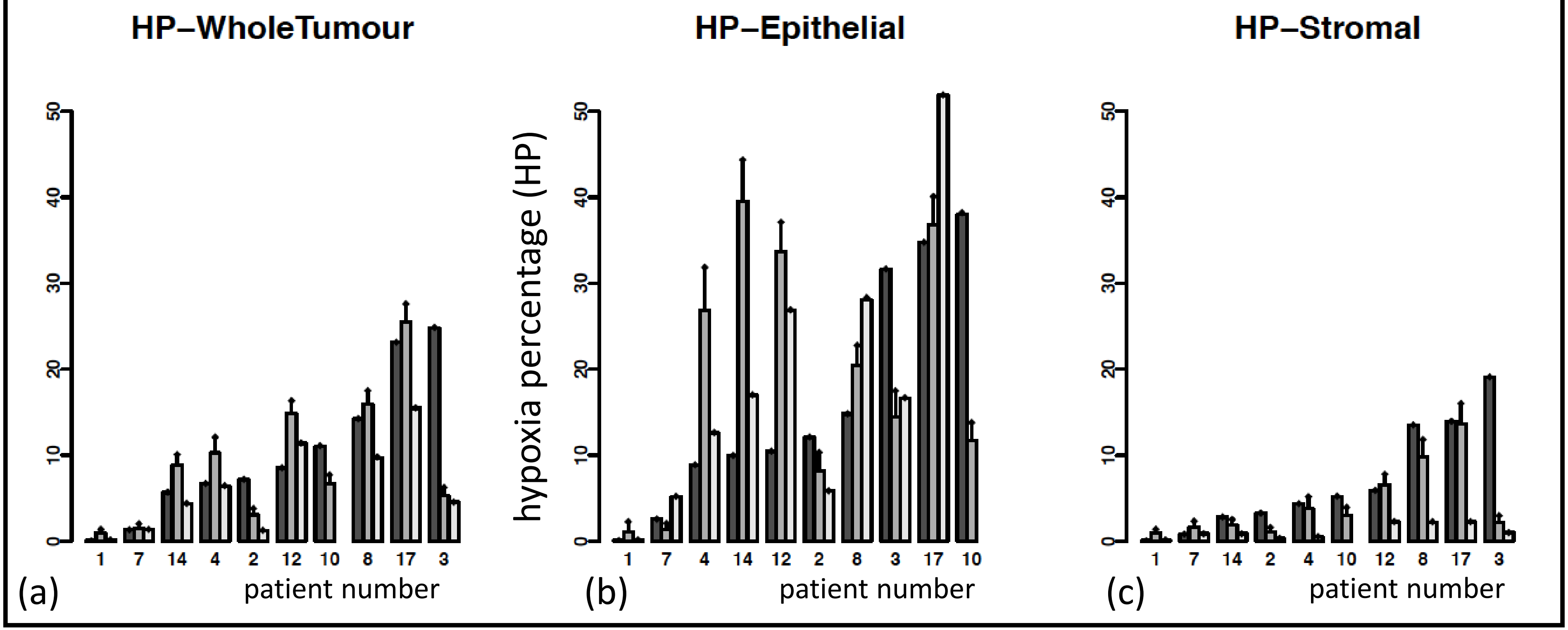

	HP: Developer	HP: Genie	HP: Tissue Studio
Whole Tumour	0 to 25 %	0 to 25 %	0 to 15 %
Epithelial	0 to 38 %	0 to 40 %	0 to 52 %
Stromal	0 to 19 %	0 to 14 %	0 to 2 %

(Definiens) Developer
(Aperio) Genie
(Definiens) Tissue Studio
